# Supplementary material for: Coagulation using organic carbonates opens up a sustainable route towards regenerated cellulose films
Source: Commun Chem. 2020 Aug 13;3:116. doi: 10.1038/s42004-020-00360-7 (PMC9814763; doi:10.1038/s42004-020-00360-7)
Supplement: Supplementary file 1 — Supplementary Information [file 42004_2020_360_MOESM1_ESM.pdf]

Supplementary Information for

Coagulation using organic carbonates opens up a sustainable route towards regenerated cellulose films

Mai N. Nguyen,et al.

[1] Department Life, Light & Matter, Faculty for Interdisciplinary Research, University of Rostock, Albert-Einstein-Straße 25, 18059 Rostock, Germany

[2] School of Chemical Engineering, Hanoi University of Science and Technology, No. 1 Dai Co Viet street, 10000 Hanoi, Vietnam

[3] Department of Chemistry, University of Rostock, Albert-Einstein-Straße 3A, 18059 Rostock, Germany

[4] Leibniz Institute for Catalysis, Albert-Einstein-Straße 29a, 18059 Rostock, Germany

[5] Institute of Physics, University of Rostock, Albert-Einstein-Straße 23-24, 18059 Rostock, Germany

[6] Medical Biology and Electron Microscopy Centre, University Medicine Rostock, 18059 Rostock, Germany

Corresponding author e-mail address: dirk.hollmann@uni-rostock.de

Supplementary Figure 1.

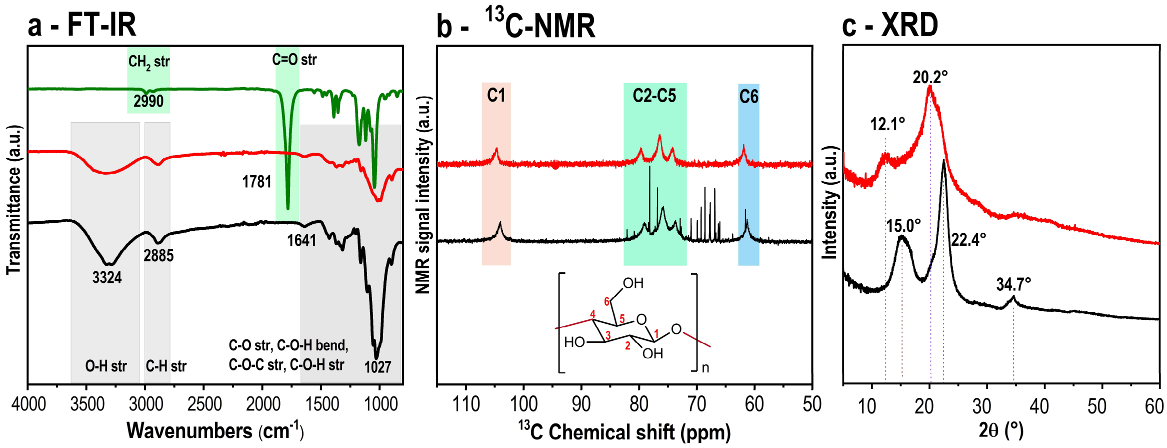

Supplementary Figure 1. Structural and chemical characterization of RC powder. (**a**) FT-IR spectra of the microcrystalline cellulose (black), RC powder (red), and propylene carbonate (**3**, green) showing the absence of C=O stretching bond and C-O of the carbonate groups in RC powder; (**b**) ^{13}C NMR of cellulose and RC powder dissolved in TBPH 50 *wt.*%; (**c**) XRD patterns of crystalline cellulose (cellulose I) and RC powder (cellulose II).

Supplementary Figure 2.

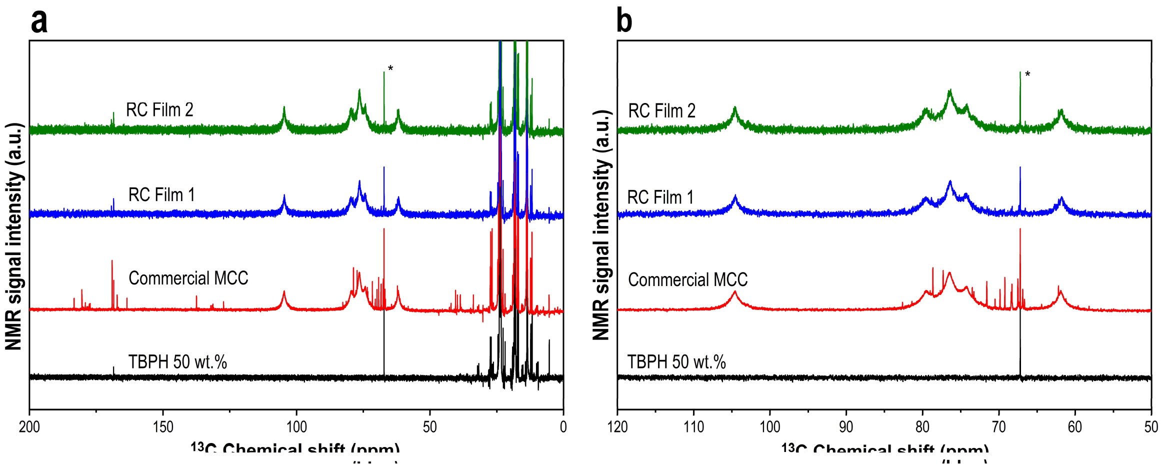

Supplementary Figure 2. ^{13}**C NMR spectra of the produced RC film 1 and 2. (a)** Overall spectra; **(b)** Zoom of the cellulose region. The signals in the range of 0-40 ppm as well as 172 ppm are impurities from the commercial TPBH, see Supplementary Figure 5. The internal standard (dioxane) is tagged with a *.

Supplementary Figure 3.

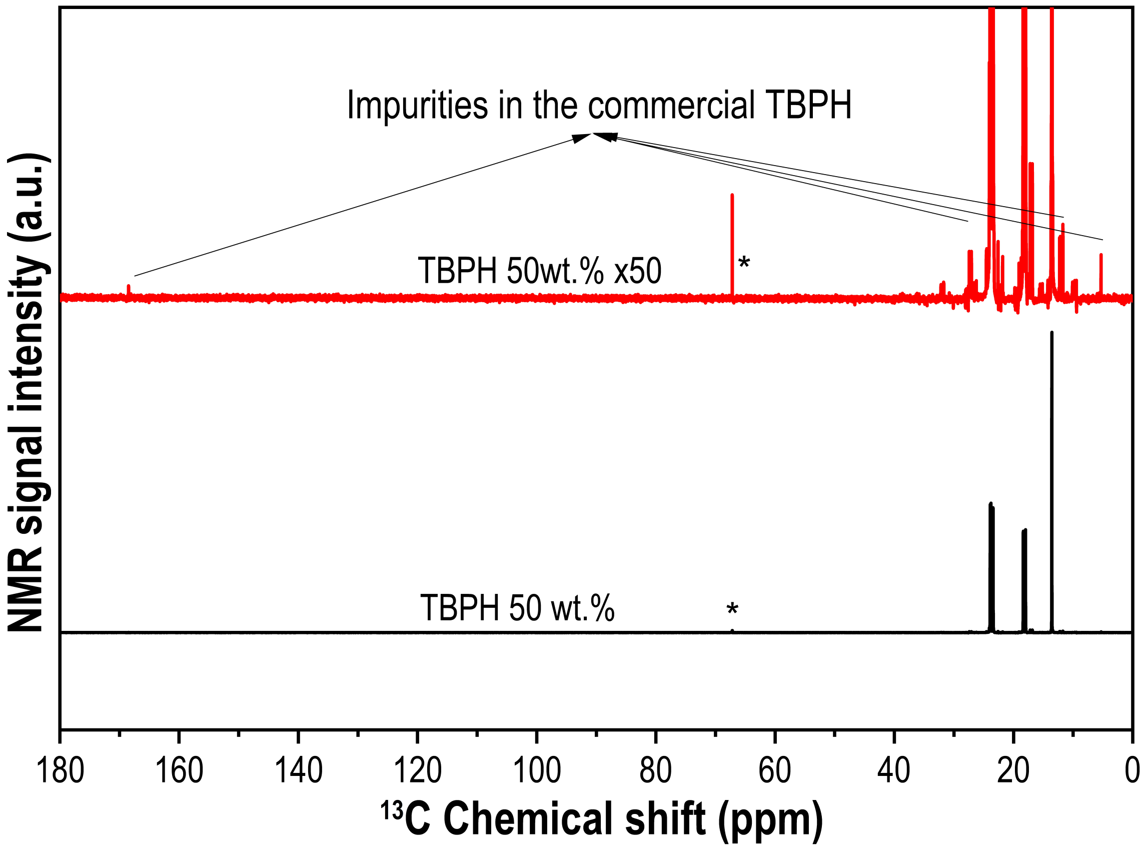

Supplementary Figure 3. ^{13}C NMR spectra of TBPH to visualize the traces of impurities in TBPH. Since TBPH is the solvent for cellulose, these impurities are always seen in the ^{13}C NMR spectra of cellulose. The internal standard (dioxane) is tagged with a *.

Supplementary Figure 4.

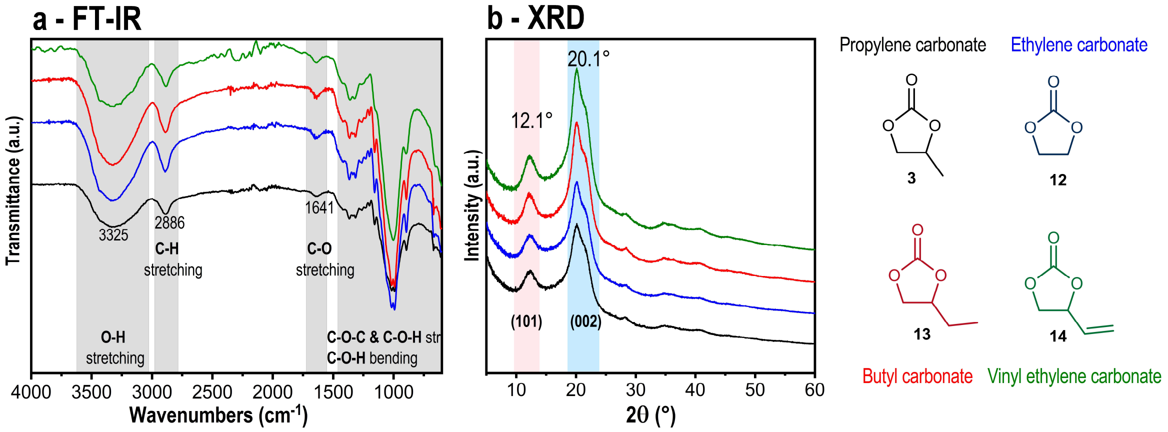

Supplementary Figure 4. FT-IR spectra and XRD patterns of regenerated cellulose powder with different kind of organic carbonate. (a) FT-IR spectra; **(b)** XRD patterns. The wavenumbers of ~3325 cm^{-1} were identified as the O−H hydroxyl group stretching vibration. The band at 2885 cm^{-1} belongs to the asymmetric stretching vibration of C-H in a pyranoid ring, 1641 cm^{-1} of the absorbed water. 1426 cm^{-1} is assigned to the CH_2 symmetric bending stretching. The band at 1160 cm^{-1} and 1027 cm^{-1} can be assigned to the C-O stretching of the C-OH deformation as well as the C-OH stretching.

Supplementary Figure 5.

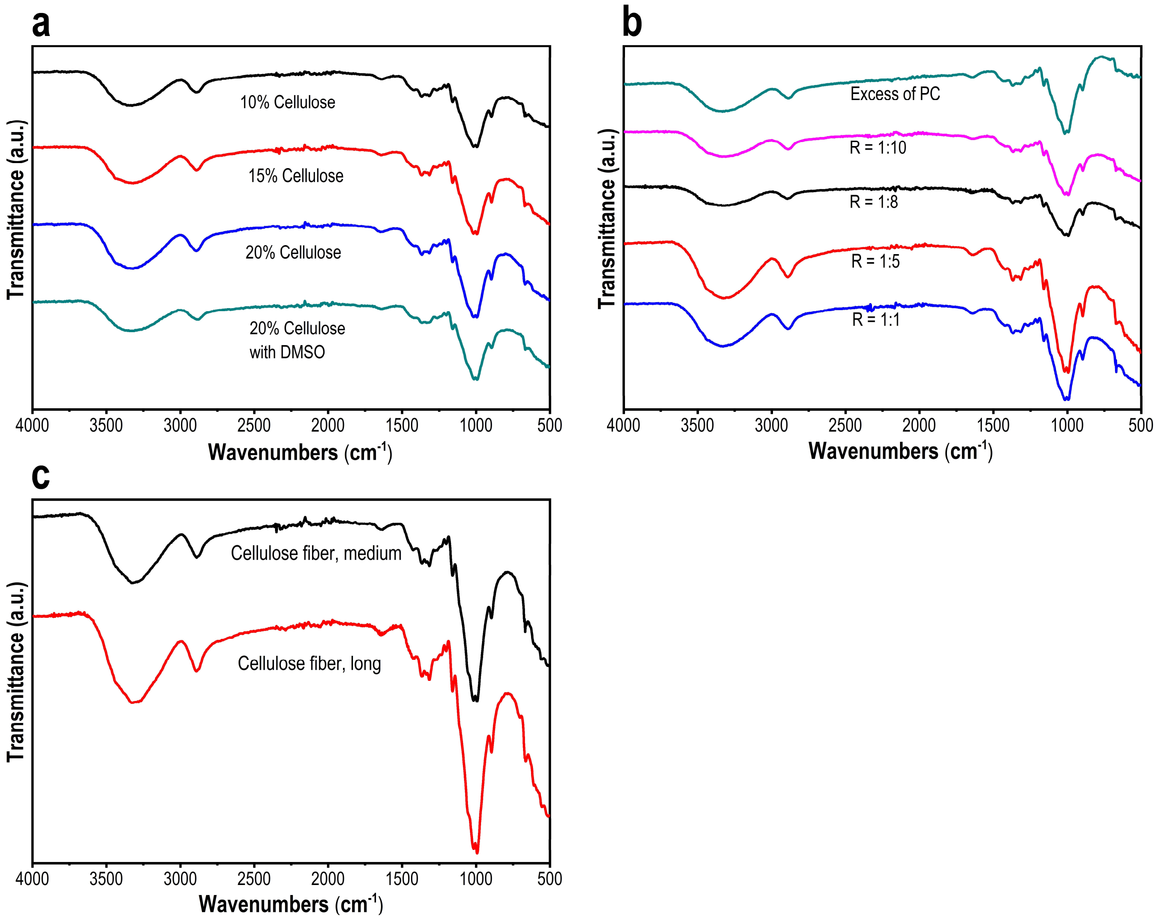

Supplementary Figure 5. FT-IR spectra of RC films with (a) different amount of cellulose (10 *wt.*%, 15 *wt.*%, 20 *wt.*%); **(b)** different ratio of **3**; **(c)** different kind of cellulose material.

Supplementary Figure 6.

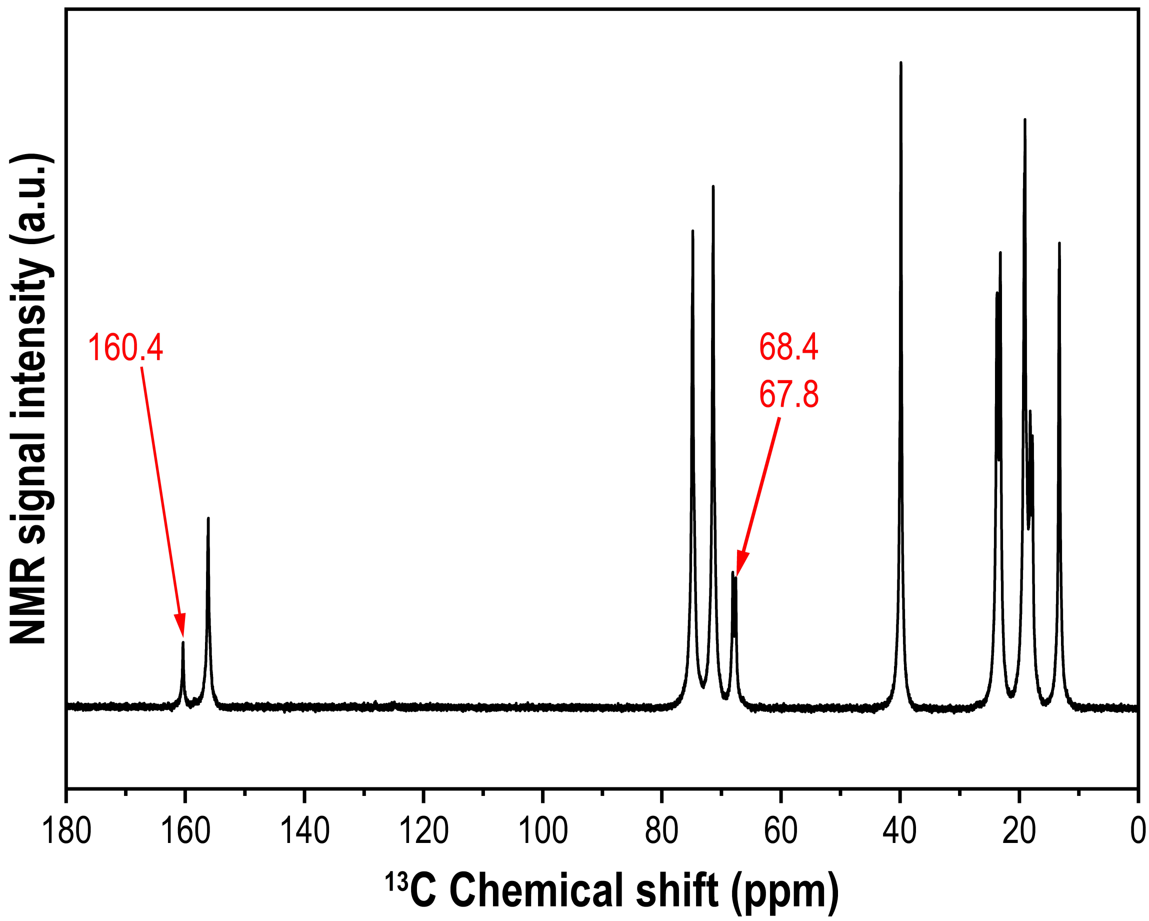

Supplementary Figure 6. ^{13}C NMR spectra of the *in-situ* reaction mixture after addition of propylene carbonate to the TBPH/Cellulose solution. The signals comprise TBPH, PC and new compounds. The solidified cellulose cannot be detected by standard solution ^{13}C NMR. The new signals are marked in red. As explained in the next pages these signals correspond to hydrogen carbonate and 1,2- propandiol (labeled as PG - propylene glycol).

Supplementary Figure 7.

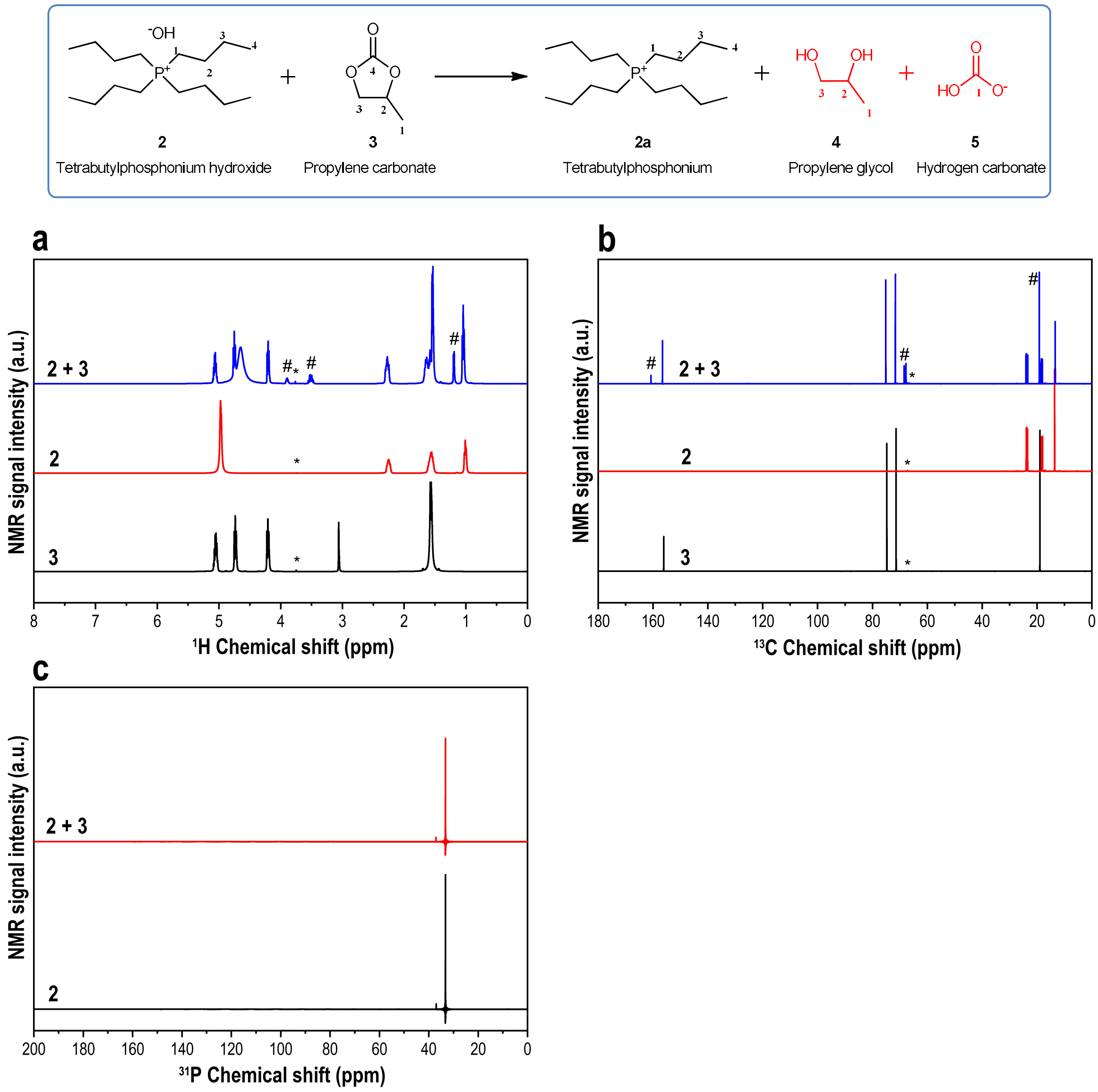

Supplementary Figure 7. ^{1}H, ^{13}C, and ^{31}P NMR spectra of propylene carbonate (3), TBPH (2) as well as the reaction mixture of both. In the mixture of **2** and **3**, new signals in ^{1}H and ^{13}C NMR (tagged as #) are detected. No shift in the ^{31}P NMR spectra (Supplementary Figure 7c) emphasize the innocent character of the $[TBP]^{+}$ (**3a**) cation. The new signals were identified as propylene glycol (**4**) and hydrogen carbonate (**5**) as described in the next section by ^{13}C DEPT, COSY, HSQC, HMBC NMR spectra. The internal standard (dioxane) is tagged with a *.

Supplementary Figure 8.

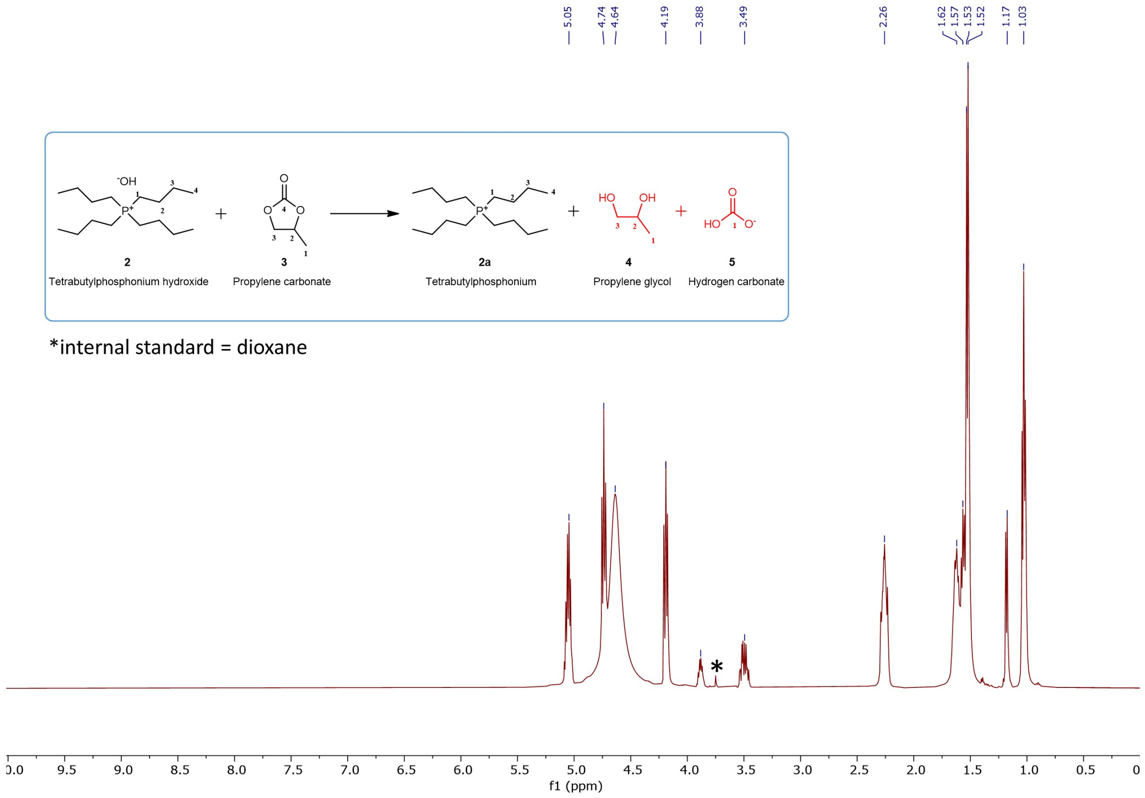

Supplementary Figure 8. ^{1}H-NMR spectra of the reaction mixture of TBPH (2) and propylene carbonate (3).

Supplementary Figure 9.

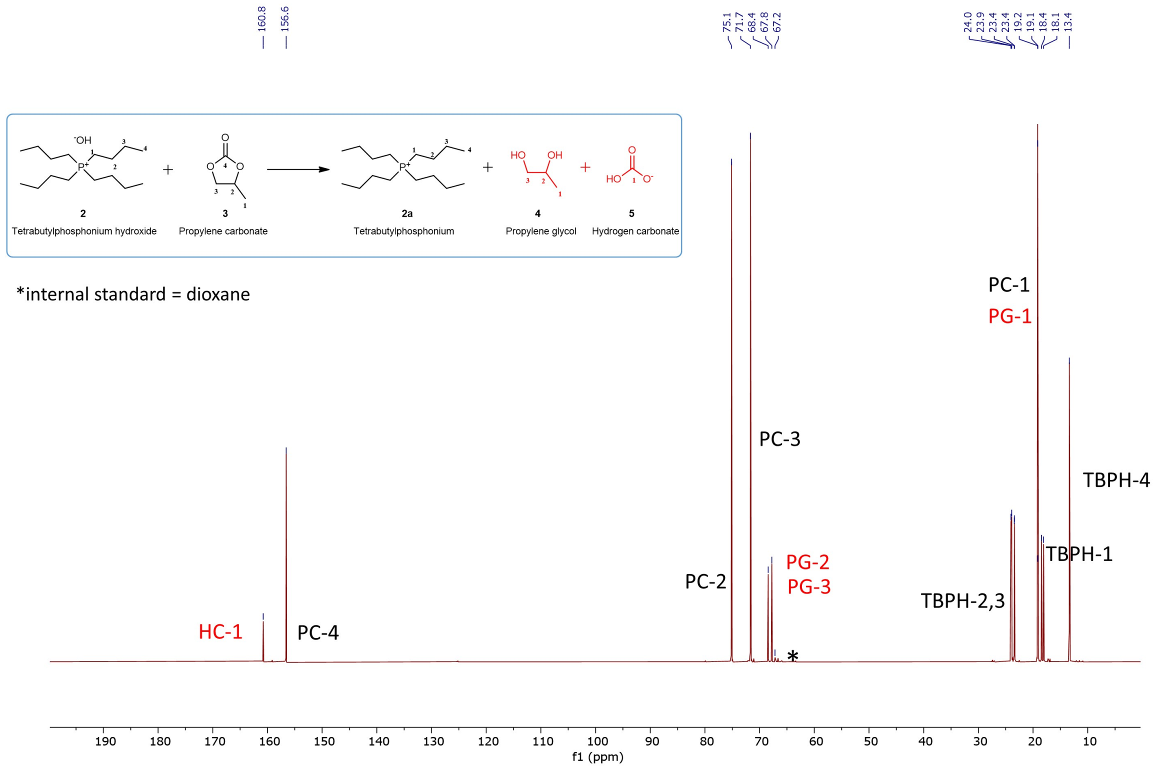

Supplementary Figure 9. ^{13}C NMR spectra of the reaction mixture of TBPH (2) and propylene carbonate (3). For better identification the signals in Figures 9-14 are labeled as followed: Tetrabutylphosphonium hydroxide (**TBPH**), Propylene carbonate (**PC**), Propylene glycol (**PG**), Hydrogen carbonate (**HC**).

Supplementary Figure 10.

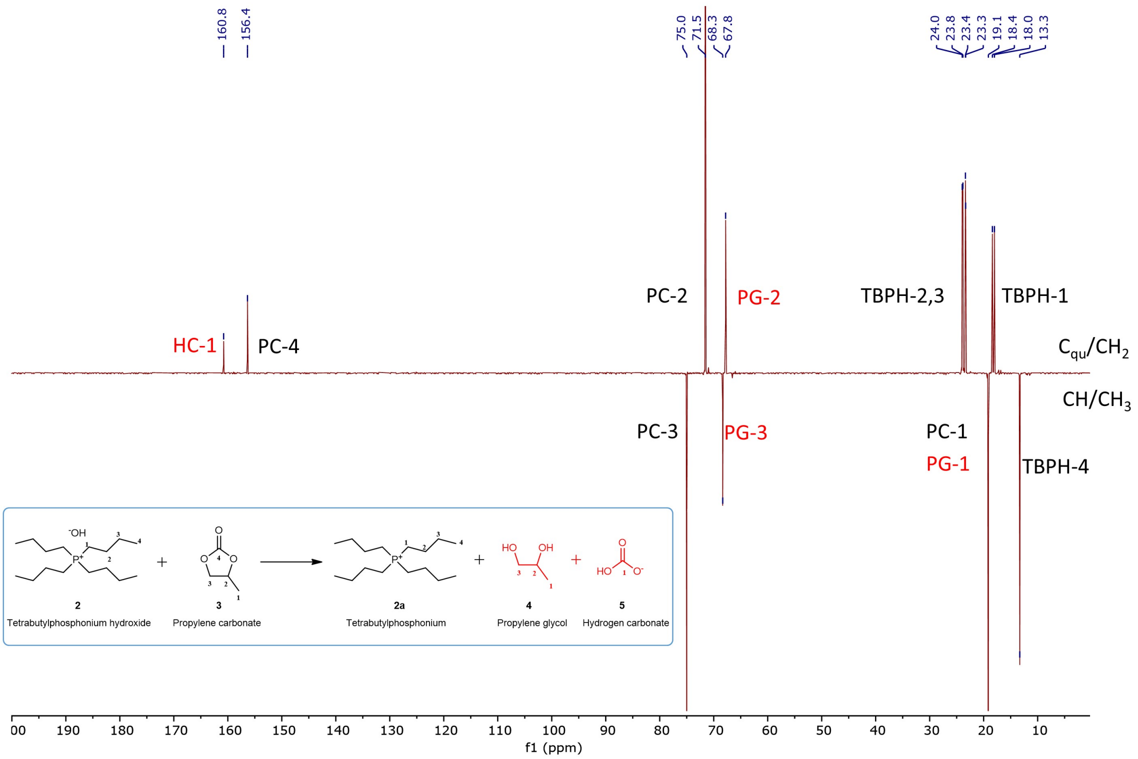

Supplementary Figure 10. ^{13}C DEPT NMR spectra of the reaction mixture of TBPH (2) and propylene carbonate (3). The signal of PC-1 and PG-1 are overlapping. Especially the identification of HC-1 and PG-1 is explained and described in the next section.

Supplementary Figure 11.

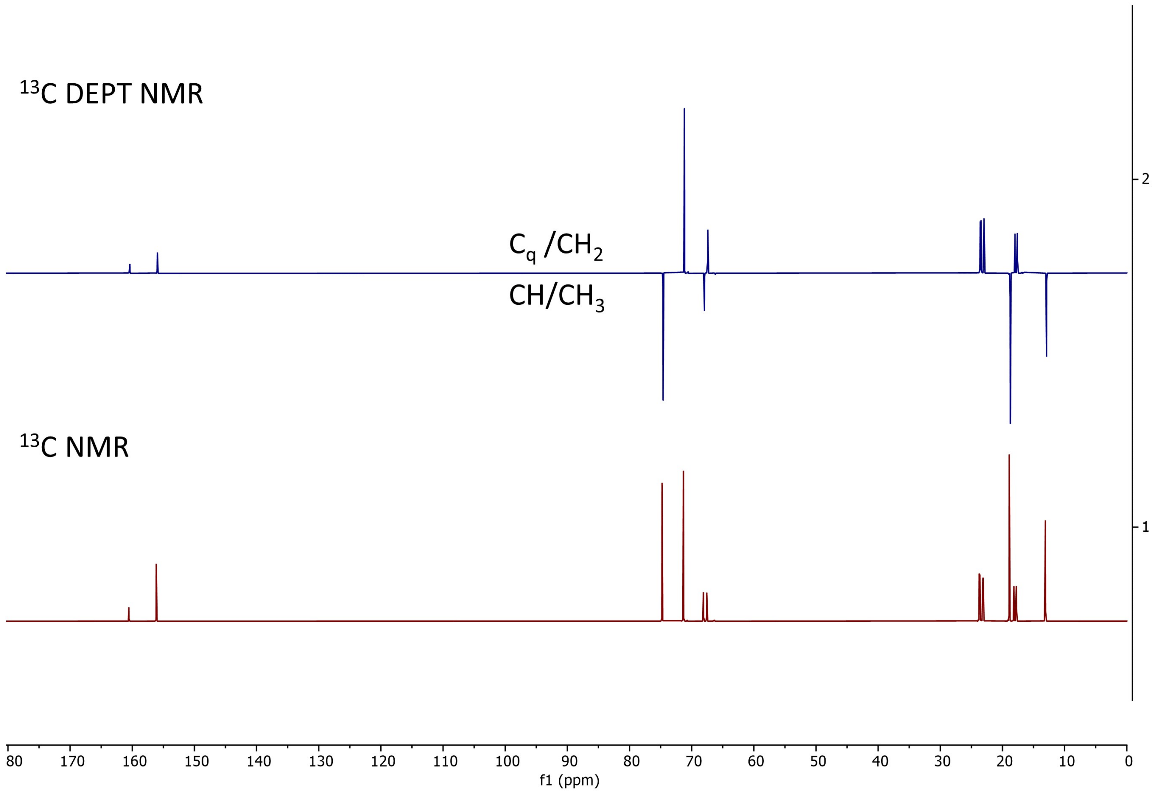

Supplementary Figure 11. Comparison ^{13}C and ^{13}C DEPT NMR spectra of the reaction mixture of TBPH (2) and propylene carbonate (3).

Supplementary Figure 12.

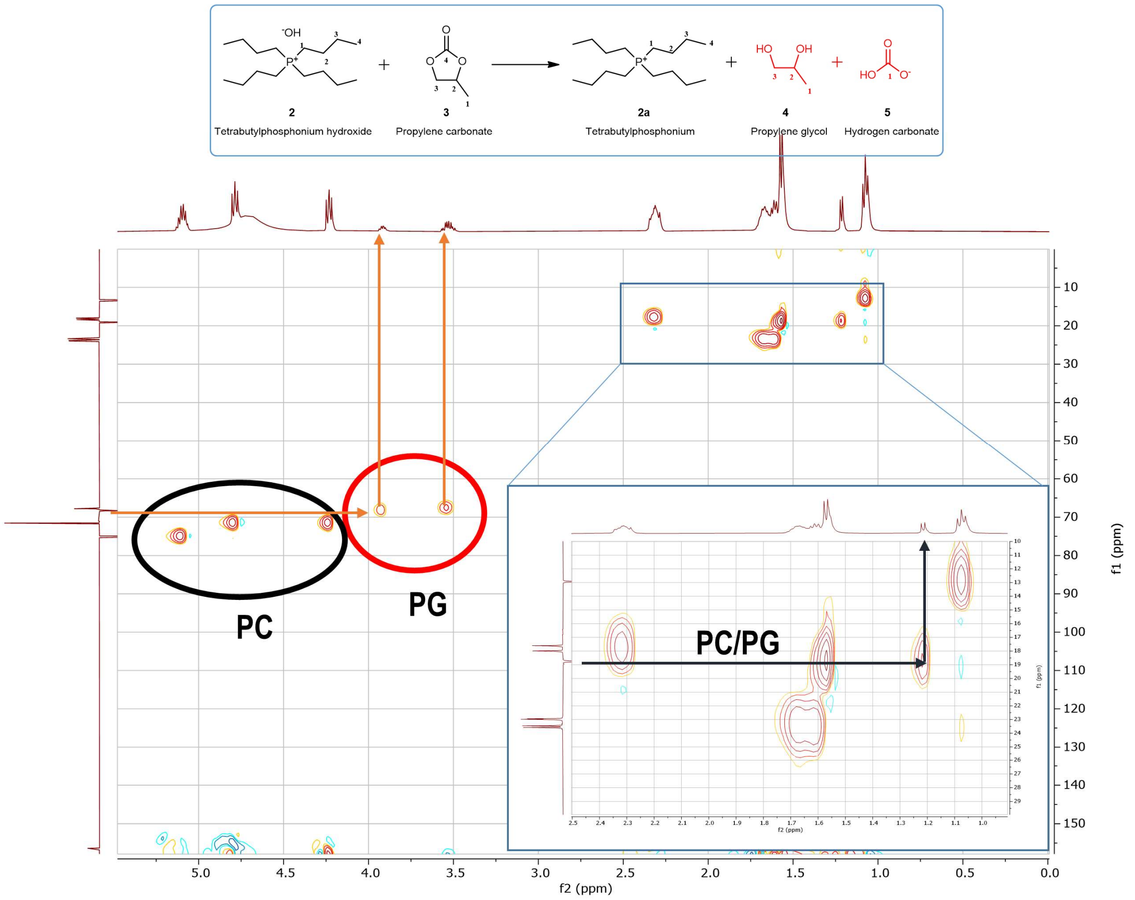

Supplementary Figure 12. ^{1}H-^{13}C HSQC NMR spectra of the reaction mixture of TBPH (2) and propylene carbonate (3). The correlation of the PG-C2 and PG-C3 to PG-C2<u>H</u> and PG-C3<u>H</u>$_2$ are highlighted in red. The carbon signal of PC-1 and PG-1 are overlapping in the ^{13}C NMR. However, since the hydrogen signals are separated, the inset shows the separated ^{1}H-^{13}C coupling of the PC-1 and PG-1. No coupling of the signal at 160.4 ppm was detected suggesting a quaternary carbon.

Supplementary Figure 13.

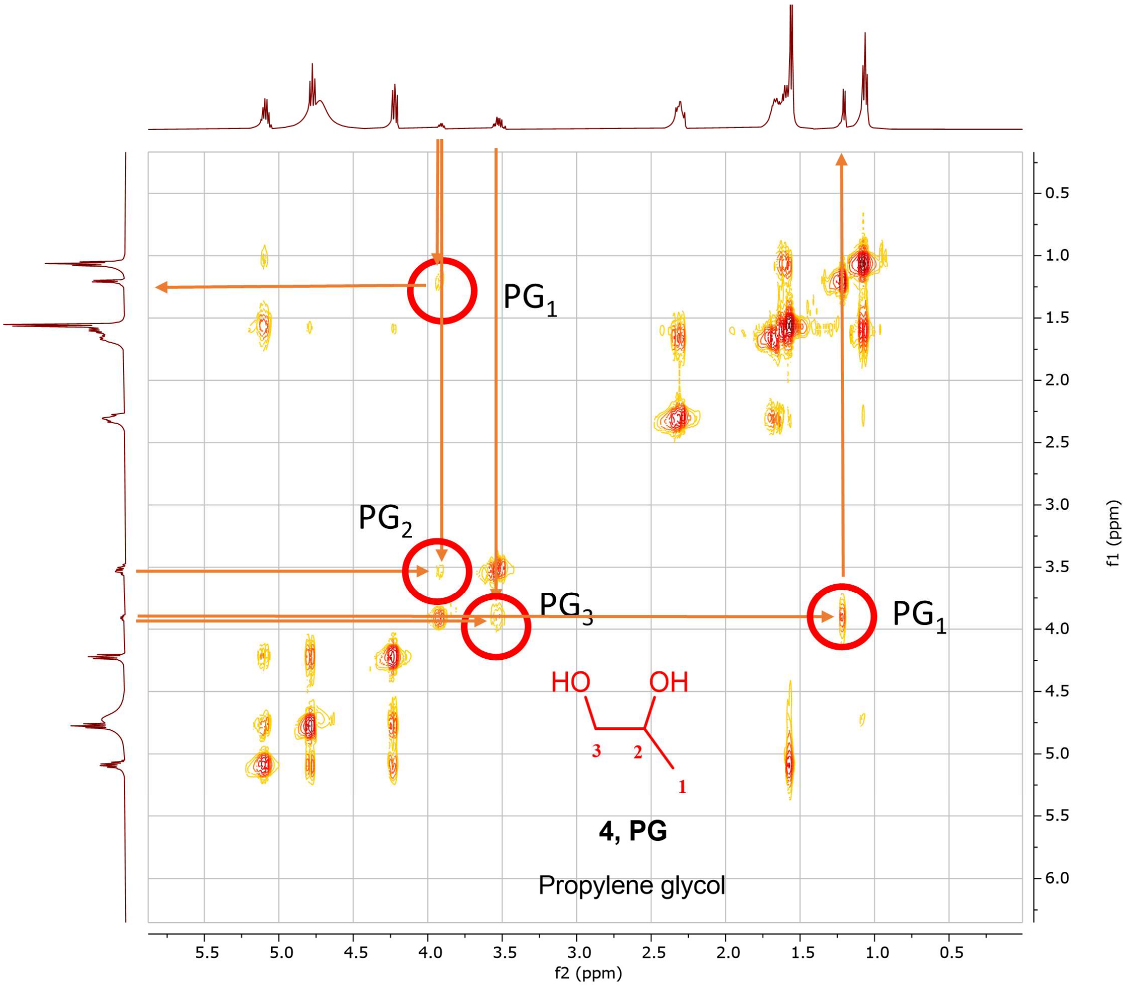

Supplementary Figure 13. ^{1}H-^{1}H COSY NMR spectra of the reaction mixture of TBPH (2) and propylene carbonate (3). Here the coupling between the hydrogen of PG-2/3 and PG-1 can be detected.

Supplementary Figure 14.

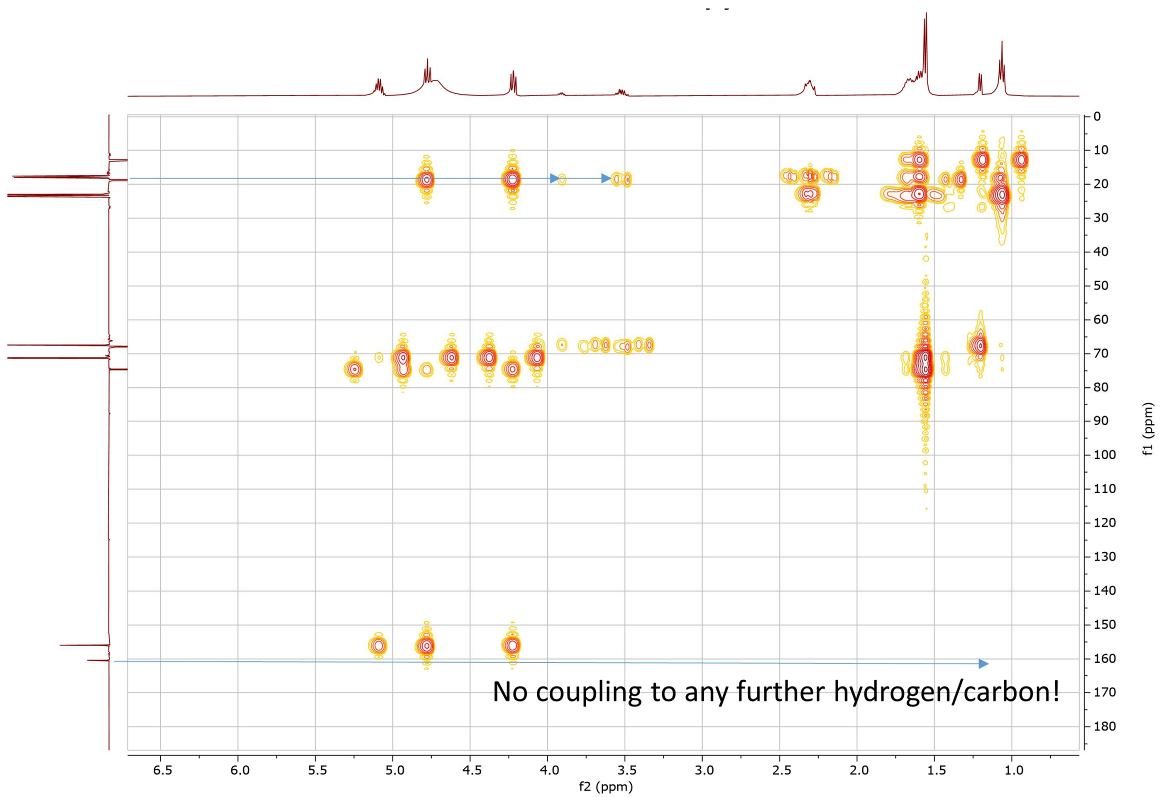

Supplementary Figure 14. ^{1}H-^{13}C HMBC NMR spectra of the reaction mixture of TBPH (2) and propylene carbonate (3). In the HMBC the coupling of carbon in one molecule can be detected. However, the signal at 160.4 ppm shows no coupling to any carbon indicating the formation of hydrogen carbonate. We proved our assumption by introduction of CO_2 in the pure aqueous TBPH solution.

Supplementary Figure 15.

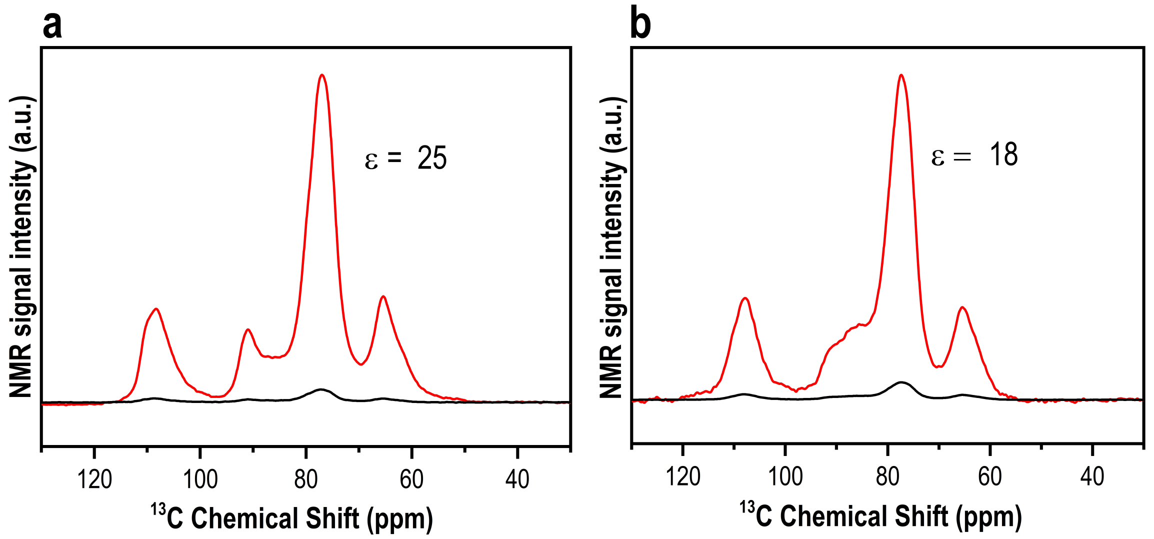

Supplementary Figure 15. DNP enhancement spectra of RC film 1 (a) and RC film 2 (b). Black without MW, red with MW. An enhancement of 25 and 18 was achieved.

Supplementary Figure 16.

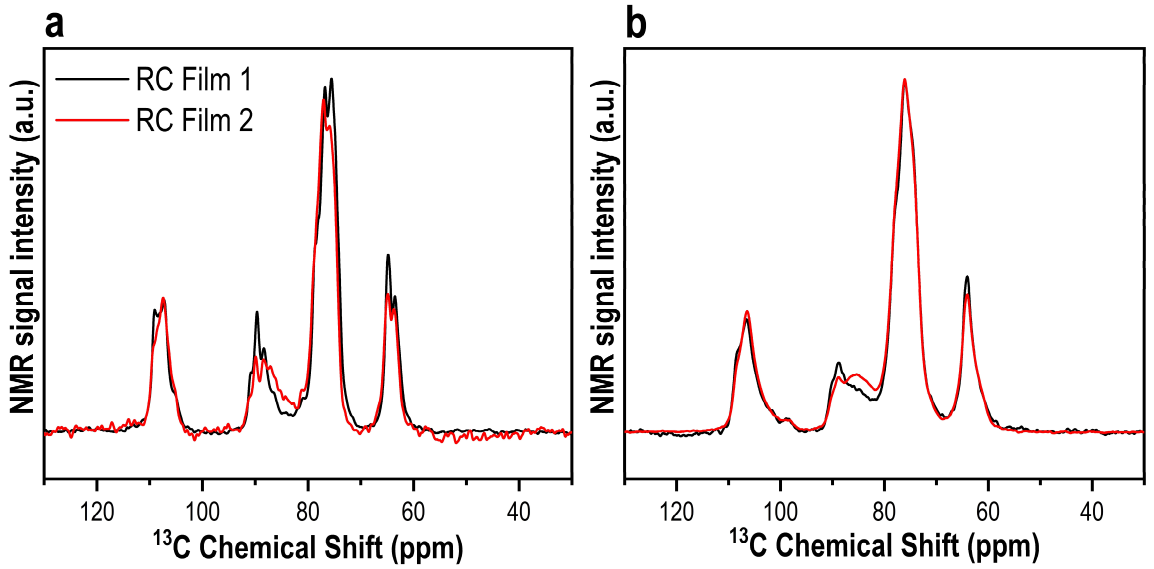

Supplementary Figure 16. ^{1}H-^{13}C Cross Polarization (CP) MAS NMR spectra of RC film 1 (black) and RC film 2 (red) in wet (a) and dried phase (b).

Supplementary Figure 17.

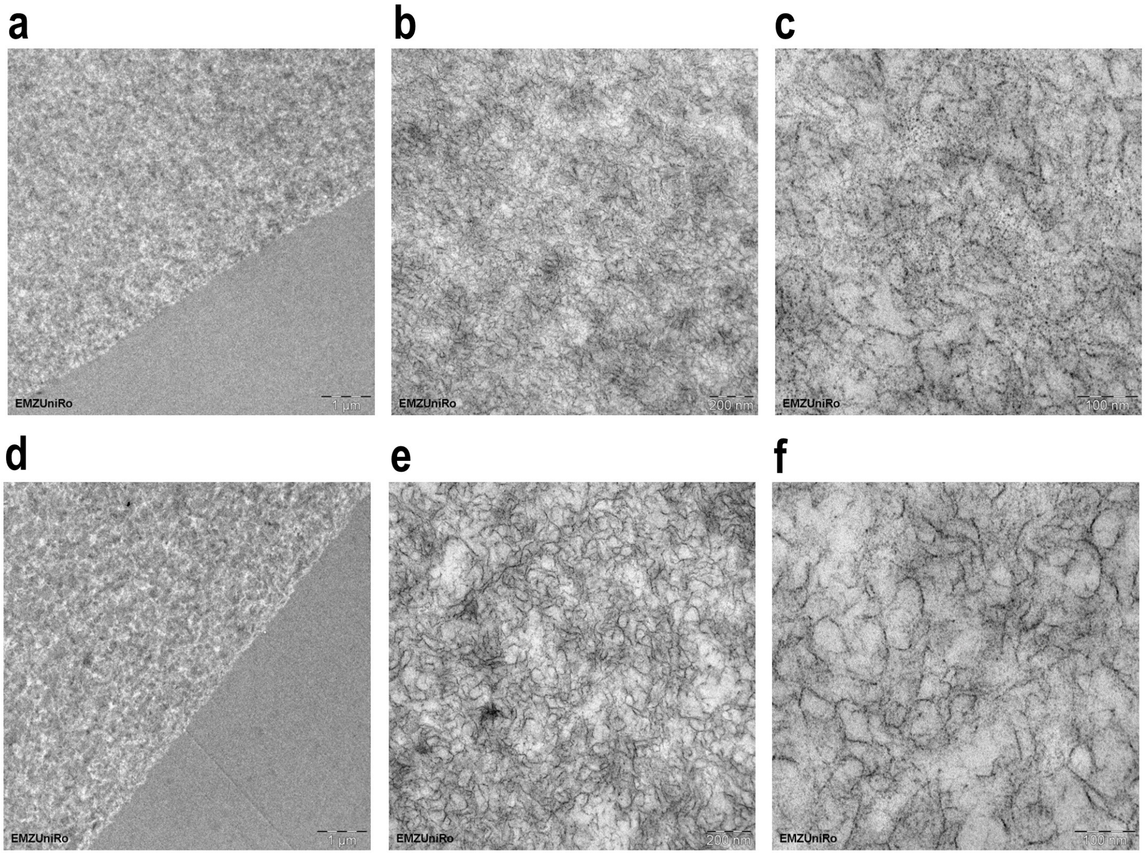

Supplementary Figure 17. TEM analysis of RC film 1. (a) Uncontrasted thin section of the film, note the smooth border of the film to the resin (bottom right), **(b)** Structure of the film with cellulose microfibrils and, **(c)** high resolution TEM of cellulose microfibrillary network after contrasting of thin sections with uranyl acetate and lead citrate.

and RC film 2. (d) Uncontrasted thin section of the film, note the smooth border of the film to the resin (bottom right), **(e)** Structure of the film with cellulose microfibrils and, **(f)** high resolution TEM of cellulose microfibrillary network after contrasting of thin sections with uranyl acetate and lead citrate.

Supplementary Figure 18.

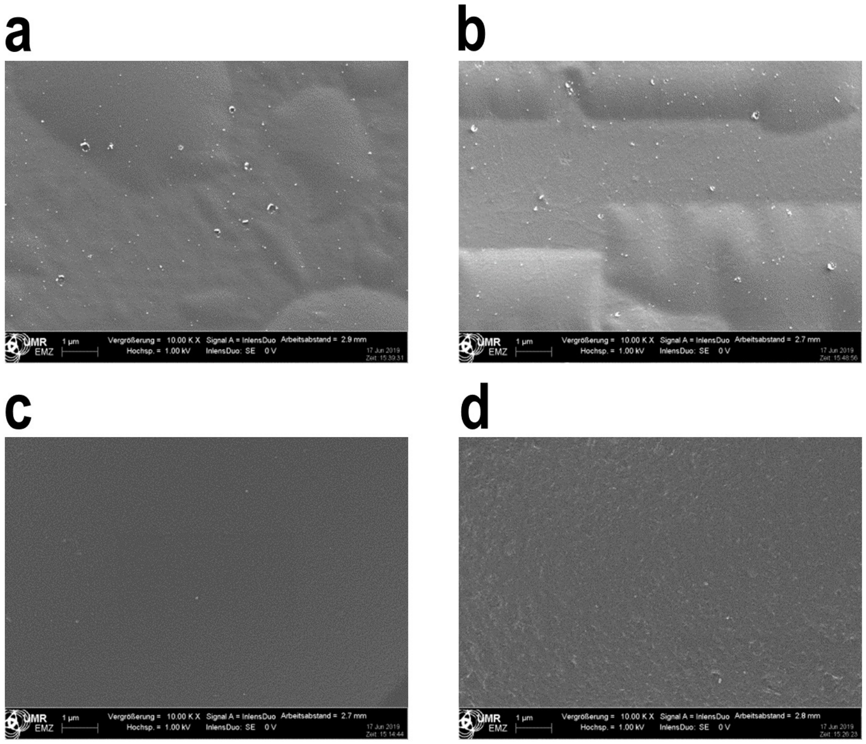

Supplementary Figure 18. SEM analysis of RC film 1 and RC film 2. (a/c) Top surface of dried film, **(b/d)** Bottom surface of dried film. Note that mild bulging visible on the otherwise smooth surfaces of RC film 1 is due to electron beam induced damage, even with a low, surface sensitive acceleration voltage decreased to 1 kV.

Supplementary Figure 19.

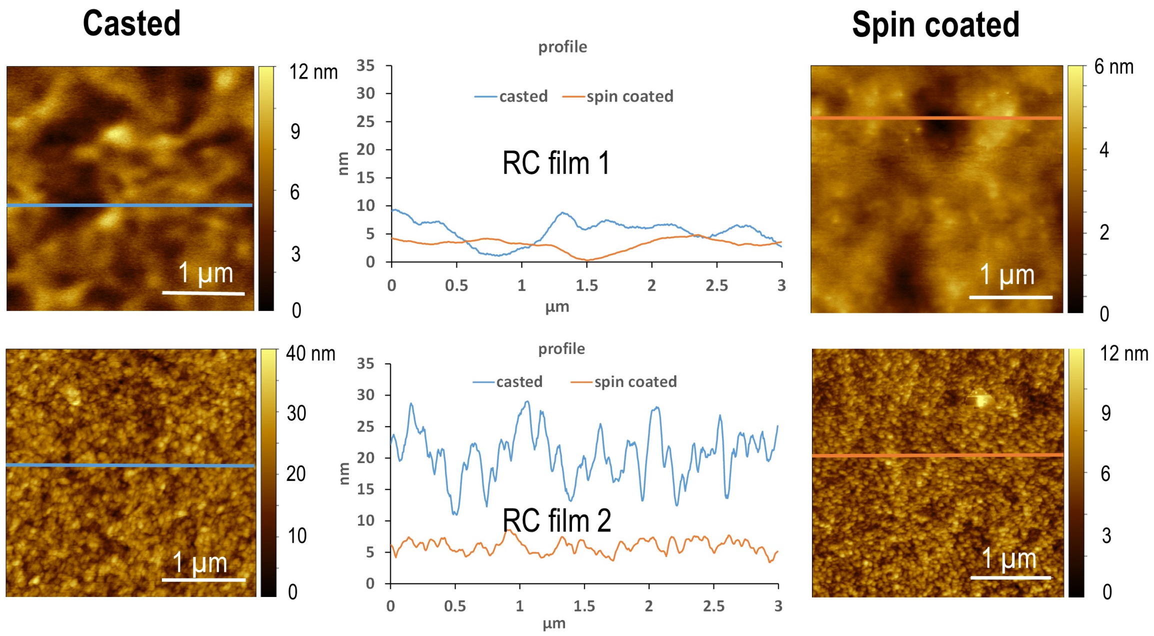

Supplementary Figure 19. AFM of casted and spin coated RC film 1 and 2. To visualize the homogeneity of the surface topology, the films were prepared by the casting technique (film thickness 500 µm, nearly perfect surface) as well as via spin coating (film thickness <1 µm, excellent surface) and dried afterwards in air.
