# Supplementary material for: Coagulation using organic carbonates opens up a sustainable route towards regenerated cellulose films
Source: Commun Chem. 2020 Aug 13;3:116. doi: 10.1038/s42004-020-00360-7 (PMC9814763; doi:10.1038/s42004-020-00360-7)
Supplement: Supplementary file 2 — Description of Additional Supplementary Files [file 42004_2020_360_MOESM2_ESM.pdf]

Description of Additional Supplementary Files

File Name: Supplementary Movie 1

Description: First, the movie explains the needs for a alternative process towards regenerated cellulose. Furthermore, our process described in our manuscript is introduced in a very general content even for non-chemist readers. Finally, the simplicity of the process is presented in our laboratory.
